# Supplementary material for: Maternal Folate Status and the Relation between Gestational Arsenic Exposure and Child Health Outcomes
Source: Int J Environ Res Public Health. 2022 Sep 9;19(18):11332. doi: 10.3390/ijerph191811332 (PMC9517145; doi:10.3390/ijerph191811332)
Supplement: Supplementary file 1 [file ijerph-19-11332-s001.zip › ijerph-1871675-supplementary.pdf]

Supplemental Material

Does Maternal Folate Status Modify the Relation Between Gestational Arsenic Exposure and Child Health Outcomes

TABLE OF CONTENTS Page #

Table S1. Central tendencies of neurodevelopmental scores and anthropometric measures on their original scales by neurodevelopmental and anthropometric profiles……………………………………………………………. 2
Table S2. Univariate statistics of maternal 1st trimester urinary arsenic species concentrations…………………... 3
Table S3. Plasma folate vitamer concentrations during the 1st trimester of pregnancy……………………. 4
Table S4. Maternal self-reported folic acid supplement consumption by dose during the 1st trimester of pregnancy…… 5
Table S5. Spearman rank correlation coefficients between log_{10}-Transformed plasma folate biomarkers and urine concentrations of arsenic species with self-reported folic acid intake supplementation during pregnancy….. 6
Table S6. Median (1st and 3rd quartile) values for 1st trimester total plasma folate, folic acid intake from supplements, and urinary DMA by neurodevelopmental and anthropometric profiles…………………………….. 7
Table S7. Unadjusted and adjusted associations of neurodevelopmental and anthropometric profiles with 10-fold increase in maternal 1st trimester urinary DMA concentrations……………………………………………………. 8
Table S8. Adjusted associations of neurodevelopmental profiles with 10-fold increase 1st trimester urinary DMA concentrations, stratified by folic acid intake from supplements and terciles of average plasma folate…………… 9
Table S9. Adjusted associations of anthropometric profiles with 10-fold increase 1st trimester urinary DMA concentrations, stratified by folic acid intake from supplements and terciles of average plasma folate…………… 10
Table S10. Unadjusted and adjusted associations of individual health outcomes per 10-fold increase in maternal 1st trimester urinary arsenic species concentrations……………………………………………………………… 11
Table S11. Adjusted difference in individual child health outcomes per 10-fold increase in maternal 1st trimester urinary DMA concentrations, stratified by folic acid intake from supplements and terciles of total plasma folate.. 12
Table S12. Adjusted associations of neurodevelopmental profiles with 10-fold increase in maternal 1st trimester urinary DMA concentrations, stratified by folic acid intake from supplements and terciles of total plasma folate.. 13
Table S13. Adjusted associations of anthropometric profiles with 10-fold increase in maternal 1st trimester urinary DMA concentrations, stratified by folic acid intake from supplements and terciles of total plasma folate.. 15
Table S14. Adjusted associations of neurodevelopmental and anthropometric profiles with 10-fold increase in maternal 1st trimester urinary DMA concentrations, accounting for uncertainty in latent profile assignment…….... 17
Table S15. Adjusted associations of neurodevelopmental profiles with 10-fold increase in maternal 1st trimester urinary DMA concentrations, stratified by folic acid intake from supplements and terciles of total plasma folate, accounting for uncertainty in latent profile assignment…………………………………………………............ 18
Table S16. Adjusted associations of anthropometric profiles with 10-fold increase in maternal 1st trimester urinary DMA concentrations, stratified by folic acid intake from supplements and terciles of total plasma folate, accounting for uncertainty in latent profile assignment………………………………………………………….. 19
Figure S1. Flow chart of participant selection to final sample size……………………………………………... 20
Figure S2. Directed Acyclic Graph used to select covariates in the association between gestational arsenic exposure and child health outcomes…………………………………………………………………………... 21
Figure S3. Pearson correlation coefficients among average plasma folate biomarkers and maternal 1st trimester urinary arsenic concentrations……………………………………………………………………………… 22

Table S1. Central tendencies of neurodevelopmental scores and anthropometric measures on their original scales by neurodevelopmental and anthropometric profiles among MIREC Study Participants

Variable	Full Sample	Neurodevelopmental Classes, Mean (SD)			Anthropometric Classes, Mean (SD)		
		Non-optimal [a] (n=31)	Typical [b] (n=183)	Optimal [c] (n=150)	Low Adiposity [d] (n=41)	Average Adiposity [e] (n=218)	Excess Adiposity [f] (n=105)
Neurodevelopmental Assessments [g]							
WPPSI: FSIQ	108 (14)	98 (19)	107 (13)	111 (12)	108 (17)	108 (13)	108 (14)
SRS T-score	45 (6.1)	55 (9.0)	47 (4.7)	42 (3.7)	45 (8.3)	45 (5.8)	46 (5.7)
BASC: Externalizing Problems	50 (7.9)	65 (7.0)	53 (4.9)	44 (4.4)	50 (8.8)	50 (8.1)	50 (7.1)
BASC: Internalizing Problems	52 (8.2)	61 (9.2)	54 (7.3)	48 (6.4)	53 (8.7)	52 (8.1)	52 (8.3)
BASC: Behavior Symptoms Index	51 (6.6)	64 (4.5)	53 (3.3)	45 (3.5)	51 (7.9)	51 (6.4)	51 (6.3)
BASC: Adaptive Skills	55 (7.1)	48 (9.3)	54 (6.2)	59 (5.7)	56 (7.6)	55 (6.8)	55 (7.6)
Anthropometric Measures							
BMI (kg/m^2)	16.2 (1.33)	16.2 (1.29)	16.2 (1.39)	16.1 (1.26)	15.3 (0.96)	15.7 (0.92)	17.5 (1.22)
Head Circumference (cm)	50.5 (1.50)	50.5 (1.31)	50.5 (1.49)	50.4 (1.56)	50.2 (1.38)	50.1 (1.44)	51.3 (1.32)
Waist Circumference (cm)	50.0 (3.89)	50.0 (4.17)	50.1 (4.10)	50.0 (3.58)	43.5 (2.35)	49.4 (2.13)	53.9 (2.86)
Hip Circumference (cm)	51.2 (4.21)	50.9 (4.10)	51.0 (4.18)	51.5 (4.28)	44.5 (2.19)	50.5 (2.59)	55.4 (3.12)
Triceps Skinfold Thickness (mm)	10.8 (3.64)	9.9 (2.99)	10.8 (2.94)	11.1 (3.64)	7.5 (1.64)	10.4 (2.94)	13.0 (4.19)
Subscapular Skinfold Thickness (mm)	6.3 (2.38)	6.3 (2.61)	6.2 (2.32)	6.4 (2.39)	5.3 (1.35)	5.7 (1.85)	7.9 (2.84)

MIREC: Maternal-Infant Research on Environmental Chemicals Study, WPPSI: Wechsler Preschool and Primary Scale of Intelligence, FSIQ: Full Scale Intelligence Quotient, SRS: Social Responsiveness Scale, BASC: Behavior Assessment System for Children, BMI: Body Mass Index

[a] Neurodevelopmental Profile: Non-optimal: (n=31) are characterized by lower cognitive abilities and more problem behaviors

[b] Neurodevelopmental Profile: Typical: (n=183) are characterized by average scores on all neurodevelopmental assessments

[c] Neurodevelopmental Profile: Optimal: (n=150) are characterized by higher cognitive abilities and less behavior problems

[d] Anthropometric Profile: Low Adiposity: (n=41) are characterized be less adiposity

[e] Anthropometric Profile: Average Adiposity: (n=218) are characterized by average adiposity

[f] Anthropometric Profile: Excess Adiposity: (n=105) are characterized by increased adiposity

[g] Note, higher FSIQ scores indicate higher cognitive abilities, higher SRS T-scores indicate more traits associated with autism spectrum disorder, higher BASC Externalizing Problems scores indicate more externalizing problem behaviors, higher BASC Internalizing Problems scores indicate more internalizing problem behaviors, higher Behavioral Symptoms Index scores indicate more problem behaviors, and lower BASC Adaptive Skills scores indicate less adaptability and diminished social skills

Table S2. Univariate statistics of maternal 1st trimester urinary arsenic species concentrations (μg As/L) among MIREC Study participants

	LOD	N (% below LOD)	Range	Median	1st & 3rd Quartile
Unstandardized					
AS-III	0.01	311 (85.4)	<LOD - 16.48	0.53	0.53, 0.53
AS-V	0.01	364 (97.5)	<LOD - 6.97	0.53	0.53, 0.53
DMA	0.01	67 (18.4)	<LOD - 44.20	2.14	1.05, 4.27
MMA	0.01	341 (93.7)	<LOD - 6.07	0.53	0.53, 0.53
AsB	0.01	184 (50.5)	<LOD - 1124	0.53	0.53, 3.97
Specific Gravity Standardized					
AS-III	--	--	0.20 - 7.91	0.64	0.40, 1.06
AS-V	--	--	0.20 - 6.36	0.79	0.33, 0.91
DMA	--	--	0.64 - 24.72	2.23	1.53, 3.38
MMA	--	--	0.20 - 6.36	0.53	0.35, 0.91
AsB	--	--	0.21 - 539.42	1.38	0.64, 4.20

MIREC: Maternal-Infant Research on Environmental Chemicals Study; LOD: Limit of Detection; AS-III: Arsenite, AS-V: Arsenate, DMA: dimethylarsinic acid, MMA: monomethylarsonous acid, AsB: arsenobetaine

Table S3. Plasma folate vitamer concentrations (nmol/L) during the 1st trimester of pregnancy among MIREC Study participants [a]

	1st Trimester		
	% <LOD [a]	Median	(1st Q, 3rd Q)
Folic Acid	2.5%	2.6	(1.0, 8.6)
5-MethylTHF	0	90.8	(74.5, 105.8)
Nonmethyl Folate [b]	-	2.2	(1.5, 3.3)
Total Folate [c]	-	99.3	(80.5, 120.1)

MIREC: Maternal-Infant Research on Environmental Chemicals Study, LOD: Limit of Detection, Q: Quartile

[a] % < LOD was not calculated for summary measures

[b] Nonmethyl folates: sum of THF, 5-FormylTHF, and 5,10-MethenylTHF

[c] Total folate: sum of five folate vitamers, 5-methylTHF, folic acid, tetrahydrofolate (THF), 5-formylTHF, and 5,10-methenylTHF

Table S4. Maternal self-reported folic acid supplement consumption by dose during the 1st trimester of pregnancy among MIREC Study participants [a]

	Categories of Folic Acid Intake		
Categories	N	Median	1st Q and 3rd Q
0-<400 $\mu g/day$	19	20	0, 171
≥400 to ≤ 1000 $\mu g/day$	254	1000	1000, 1000
>1000 $\mu g/day$	91	2000	1214, 3300

MIREC: Maternal-Infant Research on Environmental Chemicals Study, Q: Quartile

[a] Daily folic acid intake from supplements was based on maternal self-report on standardized questionnaires. Supplement intake was assessed once after the 1st trimester baseline visit. 0 includes participants who indicated that they did not take folic acid-containing supplements.

Table S5. Spearman rank correlation coefficients between log_{10}-transformed plasma folate biomarkers and urinary concentrations of DMA with self-reported folic acid intake supplementation during pregnancy among MIREC Study participants [a]

	Spearman Correlation Coefficient
Folic Acid	0.07
5-MethylTHF	0.17
Nonmethyl Folates [b]	0.09
Total Folate [c]	0.17
DMA	-0.03

MIREC: Maternal-Infant Research on Environmental Chemicals Study, DMA: dimethylarsinic acid

[a] Daily folic acid intake from supplements based on maternal self-report on standardized questionnaires, note this information was collected at only one time point, after the 1st trimester baseline visit

[b] Nonmethyl folates: sum of THF, 5-FormylTHF, and 5,10-MethenylTHF

[c] Total folates: sum of five folate vitamers: 5-MethylTHF, folic acid, tetrahydrofolate [THF], 5-FormylTHF, 5,10-MethenylTHF

Table S6. Median (1st and 3rd quartile) values for 1st trimester total plasma folate, folic acid intake from supplements, and urinary DMA by neurodevelopmental and anthropometric profiles among MIREC Study participants

	Neurodevelopmental Classes			Anthropometric Classes		
	Non-optimal [a]	Typical [b]	Optimal [c]	Low Adiposity [d]	Average Adiposity [e]	Excess Adiposity [f]
Total plasma folate [g] (nmol/L)	97.1 (89.5, 108.9)	100.2 (80.3, 121.2)	99.0 (80.2, 117.9)	92.6 (80.5, 104.2)	99.3 (80.3, 120.4)	102.7 (81.3, 127.0)
Folic Acid Intake [h] ($\mu g/day$)	1000 (1000, 1000)	1000 (1000, 1000)	1000 (1000, 1100)	1000 (1000, 1100)	1000 (1000, 1021)	1000 (1000, 1100)
DMA (μg As/L)	2.12 (1.57, 2.79)	2.38 (1.56, 3.67)	2.14 (1.50, 3.18)	2.12 (1.54, 2.54)	2.23 (1.50, 3.40)	2.38 (1.59, 3.32)

MIREC: Maternal-Infant Research on Environmental Chemicals Study; DMA: dimethylarsinic acid

[a] Neurodevelopmental Profile: Non-optimal: (n=31) are characterized by lower cognitive abilities and more problem behaviors

[b] Neurodevelopmental Profile: Typical: (n=183) are characterized by average scores on all neurodevelopmental assessments

[c] Neurodevelopmental Profile: Optimal: (n=150) are characterized by higher cognitive abilities and less behavior problems

[d] Anthropometric Profile: Low Adiposity: (n=41) are characterized be less adiposity

[e] Anthropometric Profile: Average Adiposity: (n=218) are characterized by average adiposity

[f] Anthropometric Profile: Excess Adiposity: (n=105) are characterized by increased adiposity

[g] Total folates: sum of five folate vitamers: 5-MethylTHF, folic acid, tetrahydrofolate [THF], 5-FormylTHF, 5,10-MethenylTHF

[h] Daily folic acid intake from supplements based on maternal self-report on standardized questionnaires, note this information was collected at only one time point, after the 1st trimester baseline visit

Table S7. Unadjusted and adjusted associations of neurodevelopmental and anthropometric profiles with 10-fold increase in maternal 1st trimester urinary DMA concentrations among MIREC Study participants

	Unadjusted		Adjusted [a]	
	OR (95% CI)	p-value	*OR* (95% CI)	p-value
Neurodevelopmental Profiles				
Typical [b]	1.00 (reference)	--	1.00 (reference)	--
Non-Optimal [c]	0.76 (0.20, 2.93)	0.69	0.57 (0.14, 2.38)	0.44
Optimal [d]	0.58 (0.27, 2.27)	0.17	0.44 (0.19, 1.02)	0.05
Anthropometric Profiles				
Average Adiposity	1.00 (reference)	--	1.00 (reference)	--
Low Adiposity [f]	0.63 (0.19, 2.17)	0.48	0.44 (0.11, 1.71)	0.24
Excess Adiposity [g]	1.14 (0.51, 2.58)	0.75	1.15 (0.50, 2.64)	0.74

MIREC: Maternal-Infant Research on Environmental Chemicals Study, DMA: dimethylarsinic acid, OR: odds ratio

[a] Models adjusted for maternal age at delivery (continuous), maternal birth country (Canada v. foreign), prenatal multivitamin use (yes v. no), maternal depressive symptoms (continuous CES-D scores), maternal pre-pregnancy BMI (normal/underweight v. overweight v. obese), income (continuous), and parity (nulliparous v. 1 previous child v. 2+ previous children)

[b] Neurodevelopmental Profile: Typical: (n=183) are characterized by average scores on all neurodevelopmental assessments, which served as the reference group

[c] Neurodevelopmental Profile: Non-optimal: (n=31) are characterized by lower cognitive abilities and more problem behaviors

[d] Neurodevelopmental Profile: Optimal: (n=150) are characterized by higher cognitive abilities and less behavior problems

[e] Anthropometric Profile: Average Adiposity: (n=218) are characterized by average adiposity, which served as the reference group

[f] Anthropometric Profile: Low Adiposity: (n=41) are characterized be less adiposity

[g] Anthropometric Profile: Excess Adiposity: (n=105) are characterized by increased adiposity

Table S8. Adjusted [a] associations of neurodevelopmental profiles with 10-fold increase 1st trimester urinary DMA concentrations, stratified by folic acid intake from supplements and terciles of average plasma folate, among MIREC Study participants

		Typical [b]	Non-optimal [c]		Optimal [d]	
Strata of Folate	N	(reference)	OR (95% CI)	p-value	OR (95% CI)	p-value
Total Plasma Folate (nmol/L) [e]						
T1 (<87)	121	1.00	0.11 (0.00, 3.31)	0.28	0.25 (0.05, 1.25)	0.09
T2 (87 - <111)	121	1.00	0.73 (0.09, 5.35)	0.75	0.71 (0.16, 3.09)	0.65
T3 (≥111)	122	1.00	0.92 (0.04, 22)	0.96	0.41 (0.09, 1.80)	0.24
Interaction term p-value			0.80		0.07	
Folic Acid Intake from Supplements $(\mu g/day)$ [f, g]						
>400-1000 (meets)	254	1.00	0.34 (0.05, 2.21)	0.26	0.35 (0.13, 0.98)	0.05
>1000 (exceeds)	91	1.00	3.85 (0.06, 256)	0.52	0.98 (0.15, 6.30)	0.98
Interaction term p-value			0.71		0.21	

MIREC: Maternal-Infant Research on Environmental Chemicals Study, DMA: dimethylarsinic acid, BMI: Body Mass Index, OR: Odds Ratio

[a] Models adjusted for maternal age at delivery (continuous), maternal birth country (Canada v. foreign), prenatal multivitamin use (yes v. no), maternal depressive symptoms (continuous CES-D scores), maternal pre-pregnancy BMI (normal/underweight v. overweight v. obese), income (continuous), and parity (nulliparous v. 1 previous child v. 2+ previous children).

[b] Neurodevelopmental Profile: Typical: (n=183) are characterized by average scores on all neurodevelopmental assessments, serves as the reference group

[c] Neurodevelopmental Profile: Non-optimal: (n=31) are characterized by lower cognitive abilities and more problem behaviors.

[d] Neurodevelopmental Profile: Optimal: (n=150) are characterized by higher cognitive abilities and less behavior problems.

[e] Total folate: sum of five folate vitamers, 5-methylTHF, folic acid, tetrahydrofolate (THF), 5-formylTHF, and 5,10-methenylTHF

[f] Daily folic acid intake from supplements based on maternal self-report on standardized questionnaires, note this information was collected at only one time point, after the 1st trimester baseline visit

[g] Note, we excluded participants who did not meet daily recommendations for folic acid intake from supplements due to small sample size, resulting in a subsample of n=345 participants. Within this subsample, n=171 participants were included in the Typical neurodevelopmental profile, n=30 were included in the Non-optimal profile, and n=144 were included in the Optimal profile

Table S9. Adjusted [a] associations of anthropometric profiles with 10-fold increase 1st trimester urinary DMA concentrations, stratified by folic acid intake from supplements and terciles of average plasma folate, among MIREC Study participants

Strata of Folate	N	Average Adiposity [b] (reference)	Low Adiposity [c] OR (95% CI)	Low Adiposity [c] p-value	Excess Adiposity [d] OR (95% CI)	Excess Adiposity [d] p-value
Total Plasma Folate (nmol/L) [e]						
T1 (<87)	121	1.00	0.62 (0.05, 7.57)	0.71	0.39 (0.07, 2.28)	0.30
T2 (87 - <111)	121	1.00	0.20 (0.02, 2.24)	0.19	2.37 (0.56, 10)	0.24
T3 (≥111)	122	1.00	0.11 (0.00, 8.37)	0.32	1.86 (0.39, 8.97)	0.44
Interaction term p-value			0.17		0.62	
Folic Acid Intake from Supplements ($\mu g/day$) [f, g]						
>400-1000 (meets)	254	1.00	0.73 (0.15, 3.51)	0.70	0.81 (0.30, 2.19)	0.67
>1000 (exceeds)	91	1.00	0.16 (0.01, 4.48)	0.29	15 (1.38, 157)	0.03
Interaction term p-value			0.94		0.36	

MIREC: Maternal-Infant Research on Environmental Chemicals Study, DMA: dimethylarsinic acid, BMI: Body Mass Index, OR: Odds Ratio

[a] Models adjusted for maternal age at delivery (continuous), maternal birth country (Canada v. foreign), prenatal multivitamin use (yes v. no), maternal depressive symptoms (continuous CES-D scores), maternal pre-pregnancy BMI (normal/underweight v. overweight v. obese), income (continuous), and parity (nulliparous v. 1 previous child v. 2+ previous children).

[b] Anthropometric Profile: Average Adiposity (n=218) are characterized by average adiposity, serves as the reference group

[c] Anthropometric Profile: Low Adiposity: (n=41) are characterize by less adiposity

[d] Anthropometric Profile: Excess Adiposity (n=105) are characterized by increased adiposity.

[e] Total folate: sum of the five folate vitamers, 5-methylTHF, folic acid, tetrahydrofolate (THF), 5-formylTHF, and 5,10-methenylTHF

[f] Daily folic acid intake from supplements based on maternal self-report on standardized questionnaires, note this information was collected at only one time point, after the 1st trimester baseline visit

[g] Note, we excluded participants who did not meet daily recommendations for folic acid intake from supplements due to small sample size, resulting in a subsample of n=345 participants. Within this subsample, n=203 participants were included in the Average Adiposity anthropometric profile, n=40 were included in the Low Adiposity profile, and n=102 were included in the Excess Adiposity profile.

Table S10. Unadjusted and adjusted differences in individual health outcomes per 10-fold increase in maternal 1st trimester urinary DMA concentrations among MIREC Study participants [a,b]

	Unadjusted	Adjusted
	β (95% CI)	β (95% CI)
WPPSI-FSIQ	4.0 (-1.0, 9.0)	3.1 (-2.0, 8.1)
SRS-T scores	0.5 (-1.7, 2.7)	0.5 (-1.7, 2.7)
BASC Externalizing	0.1 (-2.7, 3.0)	0.4 (-2.4, 3.3)
BASC Internalizing	3.2 (0.2, 6.1)	3.2 (0.3, 6.1)
BASC Behavior Symptom Index	0.6 (-1.8, 3.0)	0.9 (-1.4, 3.3)
BASC Adaptive Skills	0.4 (-2.1, 3.0)	-0.4 (-2.9, 2.1)
BMI (kg/m^2)	0.0 (-0.5, 0.5)	0.1 (-0.4, 0.5)
Waist Circumference (cm)	0.0 (-1.4, 1.4)	0.0 (-1.4, 1.4)
Hip Circumference (cm)	0.7 (-0.8, 2.3)	0.4 (-1.0, 1.9)
Head Circumference (cm)	0.5 (-0.1, 1.0)	0.3 (-0.2, 0.9)
Triceps Skinfold (mm)	0.9 (-0.4, 2.3)	1.2 (-0.1, 2.5)
Subscapular Skinfold (mm)	0.3 (-0.6, 1.1)	0.2 (-0.6, 1.1)

MIREC: Maternal-Infant Research on Environmental Chemicals Study, DMA: dimethylarsinic acid, WPPSI: Wechsler Preschool and Primary Scale of Intelligence, FSIQ: Full Scale Intelligence Quotient, SRS: Social Responsiveness Scale, BASC: Behavior Assessment System for Children, BMI: Body Mass Index

[a] Adjusted for: maternal age (continuous), maternal country of origin (Canadian born v. foreign born), prenatal vitamin intake (yes v. no), household income (continuous), pre-pregnancy BMI (normal and under, overweight, obese), parity (nulliparous, 1 prior child, 2 or more prior children), maternal depressive symptoms (continuous CES scores) *For anthropometric outcomes, models were also adjusted for child sex and age (in months)

[b] Note, higher FSIQ scores indicate higher cognitive abilities, higher SRS T-scores indicate more traits associated with autism spectrum disorder, higher BASC Externalizing Problems scores indicate more externalizing problem behaviors, higher BASC Internalizing Problems scores indicate more internalizing problem behaviors, higher Behavioral Symptoms Index scores indicate more problem behaviors, and lower BASC Adaptive Skills scores indicate less adaptability and diminished social skills

Table S11. Adjusted difference in individual child health outcomes per 10-fold increase in maternal 1st trimester urinary DMA concentrations, stratified by folic acid intake from supplements and terciles of total plasma folate among MIREC Study participants [a, b]

	Terciles of Total Plasma Folate (nmol/L) [c]				Categories of Folic Acid Intake from Supplements (μg/day) [d, e]		
	T1 (<87)	T2 (87 - <121)	T3 (≥111)	Interaction Term	>400-1000	>1000	Interaction Term
	β (95% CI)	β (95% CI)	β (95% CI)	p-value	β (95% CI)	β (95% CI)	p-value
WPPSI-FSIQ	5.7 (-3.2, 14.7)	5.1 (-3.6, 13.9)	0.8 (-8.5, 10.2)	0.68	2.5 (-3.5, 8.5)	-1.6 (-14.7, 11.6)	0.28
SRS-T scores	0.8 (-2.8, 4.4)	0.0 (-4.5, 4.5)	0.5 (-3.0, 4.1)	0.36	-1.0 (-3.6, 1.6)	3.2 (-1.6, 7.9)	0.61
BASC Externalizing	0.9 (-4.4, 6.1)	-1.2 (-6.0, 3.6)	0.7 (-4.6, 5.9)	0.84	0.8 (-2.6, 4.2)	-0.1 (-8.1, 7.9)	0.52
BASC Internalizing	2.6 (-2.5, 7.7)	3.6 (-2.0, 9.2)	4.0 (-0.9, 9.0)	0.01	5.0 (1.5, 8.5)	-1.2 (-8.7, 6.4)	0.39
BASC Behavior Symptom Index	0.7 (-3.8, 5.2)	-0.6 (-5.0, 3.7)	2.4 (-1.6, 6.4)	0.39	1.2 (-1.6, 3.9)	-0.4 (-7.2, 6.3)	0.52
BASC Adaptive Skills	-2.2 (-6.2, 1.9)	2.0 (-2.8, 6.9)	-2.0 (-6.6, 2.5)	0.64	0.5 (-2.7, 3.6)	-0.7 (-6.2, 4.8)	0.03
BMI (kg/m^2)	-0.1 (-1.2, 0.9)	-0.3 (-1.1, 0.4)	0.7 (-0.1, 1.5)	0.91	0.1 (-0.5, 0.7)	0.8 (-0.5, 2.1)	0.71
Waist Circumference (cm)	-0.9 (-3.8, 2.1)	0.7 (-1.6, 3.0)	0.9 (-1.4, 3.1)	0.60	0.4 (-1.3, 2.2)	-0.6 (-4.1, 2.9)	0.90
Hip Circumference (cm)	-1.0 (-3.9, 2.0)	1.2 (-1.3, 3.7)	1.3 (-1.3, 3.9)	0.10	1.1 (-0.7, 2.9)	-1.4 (-5.4, 2.5)	0.85
Head Circumference (cm)	0.5 (-0.6, 1.6)	0.1 (-0.7, 0.9)	0.6 (-0.3, 1.4)	0.21	0.4 (-0.3, 1.0)	0.9 (-0.4, 2.2)	0.49
Triceps Skinfold (mm)	0.1 (-2.4, 2.5)	1.9 (-0.2, 4.1)	1.9 (-0.5, 4.4)	0.89	1.3 (-0.2, 2.8)	1.0 (-2.9, 5.0)	0.09
Subscapular Skinfold (mm)	0.0 (-1.7, 1.8)	1.0 (-0.1, 2.1)	0.2 (-1.5, 1.9)	0.25	0.3 (-0.7, 1.2)	1.3 (-1.0, 3.5)	0.21

MIREC: Maternal-Infant Research on Environmental Chemicals Study, DMA: dimethylarsinic acid, WPPSI: Wechsler Preschool and Primary Scale of Intelligence, FSIQ: Full Scale Intelligence Quotient, SRS: Social Responsiveness Scale, BASC: Behavior Assessment System for Children, BMI: Body Mass Index

[a] Adjusted for: maternal age (continuous), maternal country of origin (Canadian born v. foreign born), prenatal vitamin intake (yes v. no), household income (continuous), pre-pregnancy BMI (normal and under, overweight, obese), parity (nulliparous, 1 prior child, 2 or more prior children), maternal depressive symptoms (continuous CES scores) *For anthropometric outcomes, models were also adjusted for child sex and age (in months)

[b] Note, higher FSIQ scores indicate higher cognitive abilities, higher SRS T-scores indicate more traits associated with autism spectrum disorder, higher BASC Externalizing Problems scores indicate more externalizing problem behaviors, higher BASC Internalizing Problems scores indicate more internalizing problem behaviors, higher Behavioral Symptoms Index scores indicate more problem behaviors, and lower BASC Adaptive Skills scores indicate less adaptability and diminished social skills

[c] Total folate: sum of the folate vitamers, 5-methylTHF, folic acid, tetrahydrofolate (THF), 5-formylTHF, and 5,10-methenylTHF

[d] Daily folic acid intake from supplements based on maternal self-report on standardized questionnaires, note this information was collected at only one time point, after the 1st trimester baseline visit

[e] Note, we excluded participants who did not meet daily recommendations for folic acid intake from supplements due to small sample size, resulting in a subsample of n=345 participants

Table S12. Adjusted associations of neurodevelopmental profiles with 10-fold increase in maternal 1st trimester urinary DMA concentrations, stratified by folic acid intake from supplements and terciles of total plasma folate among MIREC Study participants [a]

Strata of Folate	N	Typical [b] (reference)	Non-optimal [c] OR (95% CI)	Non-optimal [c] p-value	Optimal [d] OR (95% CI)	Optimal [d] p-value
**Primary Analyses**						
Total Plasma Folate (nmol/L) [e]						
T1 (<87)	121	1.00	0.11 (0.00, 3.31)	0.28	0.25 (0.05, 1.25)	0.09
T2 (87 - <111)	121	1.00	0.73 (0.09, 5.35)	0.75	0.71 (0.16, 3.09)	0.65
T3 (≥111)	122	1.00	0.92 (0.04, 22)	0.96	0.41 (0.09, 1.80)	0.24
Interaction term p-value			0.80		0.07	
Folic Acid Intake from Supplements ($\mu g/day$)[f, g]						
>400-1000 (meets)	254	1.00	0.34 (0.05, 2.21)	0.26	0.35 (0.13, 0.98)	0.05
>1000 (exceeds)	91	1.00	3.85 (0.06, 256)	0.52	0.98 (0.15, 6.30)	0.98
Interaction term p-value			0.71		0.21	
Total Plasma Folate (nmol/L) [e]						
**Additional adjustment for Folic Acid Intake from Supplements** [h]						
Total Plasma Folate						
T1 (<87)	121	1.00	0.12 (0.00, 9.22)	0.33	0.24 (0.05, 1.27)	0.09
T2 (87 - <111)	121	1.00	0.61 (0.08, 4.86)	0.64	0.57 (0.12, 2.62)	0.47
T3 (≥111)	122	1.00	2.44 (0.06, 99)	0.64	0.45 (0.10, 2.04)	0.30
Interaction term p-value			0.79		0.05	
**Additional Adjustment for Maternal Plasma Cotinine** [i]						
Total Plasma Folate						
T1 (<87)	121	1.00	0.07 (0.00, 5.93)	0.24	0.25 (0.05, 1.25)	0.09
T2 (87 - <111)	121	1.00	0.60 (0.08, 4.83)	0.63	0.82 (0.18, 3.65)	0.79
T3 (≥111)	122	1.00	1.14 (0.05, 39)	0.87	0.40 (0.09, 1.79)	0.23
Interaction term p-value			0.78		0.08	
Folic Acid Intake from Supplements ($\mu g/day$)						
>400-1000 (meets)	254	1.00	0.35 (0.05, 2.26)	0.27	0.35 (0.13, 0.98)	0.05
>1000 (exceeds)	91	1.00	8.41 (0.07, 1026)	0.39	0.90 (0.14, 5.91)	0.91
Interaction term p-value			0.91		0.15	
**Additional Adjustment for Caregiving Environment** [j]						
Total Plasma Folate						
T1 (<87)	121	1.00	0.06 (0.00, 29)	0.38	0.30 (0.06, 1.55)	0.15
T2 (87 - <111)	121	1.00	0.77 (0.09, 6.27)	0.81	0.98 (0.20, 4.76)	0.98
T3 (≥111)	122	1.00	0.56 (0.01, 22)	0.76	0.30 (0.06, 1.43)	0.13
Interaction term p-value			0.86		0.19	
Folic Acid Intake from						

Supplements ($\mu g/day$)						
>400-1000 (meets)	254	1.00	0.31 (0.05, 2.01)	0.22	0.33 (0.11, 0.95)	0.04
>1000 (exceeds)	91	1.00	11 (0.05, 2536)	0.38	2.22 (0.26, 19)	0.47
Interaction term p-value			0.77		0.12	

MIREC: Maternal-Infant Research on Environmental Chemicals Study, DMA: dimethylarsinic acid, BMI: Body Mass Index

[a] Models adjusted for maternal age at delivery (continuous), maternal birth country (Canada v. foreign), prenatal multivitamin use (yes v. no), maternal depressive symptoms (continuous CES-D scores), maternal pre-pregnancy BMI (normal/underweight v. overweight v. obese), income (continuous), and parity (nulliparous v. 1 previous child v. 2+ previous children).

[b] Neurodevelopmental Profile: Typical: (n=183) are characterized by average scores on all neurodevelopmental assessments, serves as the reference group

[c] Neurodevelopmental Profile: Non-optimal: (n=31) are characterized by lower cognitive abilities and more problem behaviors.

[d] Neurodevelopmental Profile: Optimal: (n=150) are characterized by higher cognitive abilities and less behavior problems.

[e] Total folate: sum of five folate vitamers, 5-methylTHF, folic acid, tetrahydrofolate (THF), 5-formylTHF, and 5,10-methenylTHF

[f] Daily folic acid intake from supplements based on maternal self-report on standardized questionnaires, note this information was collected at only one time point, after the 1st trimester baseline visit

[g] Note, we excluded participants who did not meet daily recommendations for folic acid intake from supplements due to small sample size, resulting in a subsample of n=345 participants. Within this subsample, n=171 participants were included in the Typical neurodevelopmental profile, n=30were included in the Non-optimal profile, and n=144 were included in the Optimal profile

[h] Additional adjustment for folic acid intake from supplements

[i] Additional adjustment for log_{10}-transformed maternal plasma cotinine concentrations as a proxy of smoking during gestation

[j] Additional adjustment for total Home Observation for Measurement of the Environment (HOME Inventory) scores as a measure of caregiving environment

Table S13. Adjusted associations by anthropometric profiles with 10-fold increase in maternal 1st trimester urinary DMA concentrations, stratified by folic acid intake from supplements and terciles of total plasma folate among MIREC Study participants [a]

Strata of Folate	N	Average Adiposity [b] (reference)	Low Adiposity [c] *OR* (95% CI)	Low Adiposity [c] p-value	Excess Adiposity [d] *OR* (95% CI)	Excess Adiposity [d] p-value
**Primary Analyses**						
Total Plasma Folate (nmol/L) [e]						
T1 (<87)	121	1.00	0.62 (0.05, 7.57)	0.71	0.39 (0.07, 2.28)	0.30
T2 (87 - <111)	121	1.00	0.20 (0.02, 2.24)	0.19	2.37 (0.56, 10.11)	0.24
T3 (≥111)	122	1.00	0.11 (0.00, 8.37)	0.32	1.86 (0.39, 8.97)	0.44
Interaction term p-value			0.17		0.62	
Folic Acid Intake from Supplements ($\mu g/day$) [f, g]						
>400-1000 (meets)	254	1.00	0.73 (0.15, 3.51)	0.70	0.81 (0.30, 2.19)	0.67
>1000 (exceeds)	91	1.00	0.16 (0.01, 4.48)	0.29	15 (1.38, 157)	0.03
Interaction term p-value			0.94		0.36	
**Additional adjustment for Folic Acid Intake from Supplements** [h]						
Total Plasma Folate						
T1 (<87)	121	1.00	0.83 (0.07, 11)	0.88	0.36 (0.06, 2.27)	0.28
T2 (87 - <111)	121	1.00	0.09 (0.01, 1.32)	0.08	2.23 (0.51, 9.66)	0.28
T3 (≥111)	122	1.00	0.16 (0.00, 10)	0.39	1.84 (0.37, 9.26)	0.46
Interaction term p-value			0.15		0.67	
**Additional Adjustment for Maternal Plasma Cotinine** [i]						
Total Plasma Folate						
T1 (<87)	121	1.00	0.60 (0.05, 7.63)	0.69	0.39 (0.07, 2.29)	0.30
T2 (87 - <111)	121	1.00	0.18 (0.02, 2.08)	0.17	2.23 (0.52, 9.50)	0.28
T3 (≥111)	122	1.00	0.11 (0.00, 8.37)	0.32	2.00 (0.41, 9.82)	0.39
Interaction term p-value			0.16		0.65	
Folic Acid Intake from Supplements ($\mu g/day$)						
>400-1000 (meets)	254	1.00	0.73 (0.15, 3.52)	0.70	0.81 (0.30, 2.20)	0.68
>1000 (exceeds)	91	1.00	0.12 (0.00, 3.75)	0.23	18 (1.51, 209)	0.02
Interaction term p-value			0.89		0.20	
**Additional Adjustment for Caregiving Environment** [j]						
Total Plasma Folate						
T1 (<87)	121	1.00	0.55 (0.07 6.91)	0.64	0.33 (0.05, 2.04)	0.22
T2 (87 - <111)	121	1.00	0.15 (0.01, 1.93)	0.15	1.59 (0.33, 7.42)	0.56
T3 (≥111)	122	1.00	0.10 (0.00, 11)	0.33	2.00 (0.41, 9.73)	0.39
Interaction term p-value			0.18		0.65	
Folic Acid Intake from						

Supplements ($\mu g/day$)						
>400-1000 (meets)	254	1.00	0.68 (0.14, 3.27)	0.63	0.74 (0.27, 2.03)	0.56
>1000 (exceeds)	91	1.00	0.13 (0.00, 4.17)	0.25	9.18 (0.69, 122)	0.09
Interaction term p-value			0.90		0.20	

MIREC: Maternal-Infant Research on Environmental Chemicals Study, DMA: dimethylarsinic acid, BMI: Body Mass Index

[a] Models adjusted for maternal age at delivery (continuous), maternal birth country (Canada v. foreign), prenatal multivitamin use (yes v. no), maternal depressive symptoms (continuous CES-D scores), maternal pre-pregnancy BMI (normal/underweight v. overweight v. obese), income (continuous), and parity (nulliparous v. 1 previous child v. 2+ previous children)

[b] Anthropometric Profile: Low Adiposity: (n=41) are characterized be less adiposity

[c] Anthropometric Profile: Average Adiposity: (n=218) are characterized by average adiposity

[d] Anthropometric Profile: Excess Adiposity: (n=105) are characterized by increased adiposity

[e] Total folate: sum of the five folate vitamers, 5-methylTHF, folic acid, tetrahydrofolate (THF), 5-formylTHF, and 5,10-methenylTHF

[f] Daily folic acid intake from supplements based on maternal self-report on standardized questionnaires, note this information was collected at only one time point, after the 1st trimester baseline visit

[g] Note, we excluded participants who did not meet daily recommendations for folic acid intake from supplements due to small sample size, resulting in a subsample of n=345 participants. Within this subsample, n=203 participants were included in the Average Adiposity anthropometric profile, n=40 were included in the Low Adiposity profile, and n=102 were included in the Excess Adiposity profile.

[h] Additional adjustment for folic acid intake from supplements

[i] Additional adjustment for log_{10}-transformed maternal plasma cotinine concentrations as a proxy of smoking during gestation

[j] Additional adjustment for total Home Observation for Measurement of the Environment (HOME Inventory) scores as a measure of caregiving environment

Table S14. Adjusted associations of neurodevelopmental and anthropometric profiles with maternal 1st trimester urinary DMA concentrations, accounting for uncertainty in latent profile assignment, among MIREC Study participants

	Adjusted [a]	
	OR (95% CI)	p-value
Neurodevelopmental Profiles		
Typical [b]	1.00 (reference)	--
Non-Optimal [c]	0.57 (0.11, 2.91)	0.50
Optimal [d]	0.44 (0.20, 1.00)	0.05
Anthropometric Profiles		
Average Adiposity	1.00 (reference)	--
Low Adiposity [f]	0.44 (0.13, 1.52)	0.19
Excess Adiposity [g]	1.15 (0.50, 2.67)	0.74

MIREC: Maternal-Infant Research on Environmental Chemicals Study, DMA: dimethylarsinic acid, OR: odds ratio

[a] Models adjusted for maternal age at delivery (continuous), maternal birth country (Canada v. foreign), prenatal multivitamin use (yes v. no), maternal depressive symptoms (continuous CES-D scores), maternal pre-pregnancy BMI (normal/underweight v. overweight v. obese), income (continuous), and parity (nulliparous v. 1 previous child v. 2+ previous children)

[b] Neurodevelopmental Profile: Typical: (n=183) are characterized by average scores on all neurodevelopmental assessments, which served as the reference group

[c] Neurodevelopmental Profile: Non-optimal: (n=31) are characterized by lower cognitive abilities and more problem behaviors

[d] Neurodevelopmental Profile: Optimal: (n=150) are characterized by higher cognitive abilities and less behavior problems

[e] Anthropometric Profile: Average Adiposity: (n=218) are characterized by average adiposity, which served as the reference group

[f] Anthropometric Profile: Low Adiposity: (n=41) are characterized be less adiposity

[g] Anthropometric Profile: Excess Adiposity: (n=105) are characterized by increased adiposity

Table S15. Adjusted associations of neurodevelopmental profiles with 10-fold increase in maternal 1st trimester urinary DMA concentrations, stratified by folic acid intake from supplements and terciles of total plasma folate, accounting for uncertainty in latent profile assignment, among MIREC Study participants [a]

		Typical [b]	Non-optimal [c]		Optimal [d]	
Strata of Folate	N	(reference)	OR (95% CI)	p-value	OR (95% CI)	p-value
Total Plasma Folate (nmol/L) [e]						
T1 (<87)	121	1.00	0.11 (0.00, 2.34)	0.16	0.25 (0.05, 1.31)	0.10
T2 (87 - <111)	121	1.00	0.73 (0.07, 7.28)	0.79	0.71 (0.20, 2.59)	0.61
T3 (≥111)	122	1.00	0.92 (0.12, 7.17)	0.93	0.41 (0.09, 1.89)	0.25
Interaction term p-value			0.73		0.09	
Folic Acid Intake from Supplements $(\mu g/day)$ [f, g]						
>400-1000 (meets)	254	1.00	0.34 (0.03, 3.79)	0.38	0.35 (0.13, 0.97)	0.04
>1000 (exceeds)	91	1.00	3.85 (0.25, 59)	0.31	0.98 (0.12, 7.95)	0.98
Interaction term p-value			0.82		0.14	

MIREC: Maternal-Infant Research on Environmental Chemicals Study, DMA: dimethylarsinic acid, BMI: Body Mass Index, OR: Odds Ratio

[a] Models adjusted for maternal age at delivery (continuous), maternal birth country (Canada v. foreign), prenatal multivitamin use (yes v. no), maternal depressive symptoms (continuous CES-D scores), maternal pre-pregnancy BMI (normal/underweight v. overweight v. obese), income (continuous), and parity (nulliparous v. 1 previous child v. 2+ previous children).

[b] Neurodevelopmental Profile: Typical: (n=183) are characterized by average scores on all neurodevelopmental assessments, serves as the reference group

[c] Neurodevelopmental Profile: Non-optimal: (n=31) are characterized by lower cognitive abilities and more problem behaviors.

[d] Neurodevelopmental Profile: Optimal: (n=150) are characterized by higher cognitive abilities and less behavior problems.

[e] Total folate: sum of the five folate vitamers, 5-methylTHF, folic acid, tetrahydrofolate (THF), 5-formylTHF, and 5,10-methenylTHF

[f] Daily folic acid intake from supplements based on maternal self-report on standardized questionnaires, note this information was collected at only one time point, after the 1st trimester baseline visit

[g] Note, we excluded participants who did not meet daily recommendations for folic acid intake from supplements due to small sample size, resulting in a subsample of n=345 participants. Within this subsample, n=171 participants were included in the Typical neurodevelopmental profile, n=30 were included in the Non-optimal profile, and n=144 were included in the Optimal profile

Table S16. Adjusted associations of anthropometric profiles with 10-fold increase in maternal 1st trimester urinary DMA concentrations, stratified by folic acid intake from supplements and terciles of average plasma folate, accounting for uncertainty in latent profile assignment, among MIREC Study participants [a]

Strata of Folate	N	Average Adiposity [b] (reference)	Low Adiposity [c] OR (95% CI)	Low Adiposity [c] p-value	Excess Adiposity [d] OR (95% CI)	Excess Adiposity [d] p-value
Total Plasma Folate (nmol/L) [e]						
T1 (<87)	121	1.00	0.62 (0.07, 5.11)	0.66	0.39 (0.07, 2.19)	0.29
T2 (87 - <111)	121	1.00	0.20 (0.02, 1.72)	0.14	2.37 (0.52, 11)	0.26
T3 (≥111)	122	1.00	0.12 (0.00, 12)	0.36	1.86 (0.43, 8.08)	0.41
Interaction term p-value			0.23		0.61	
Folic Acid Intake from Supplements ($\mu g/day$) [f, g]						
>400-1000 (meets)	254	1.00	0.73 (0.18, 2.96)	0.66	0.81 (0.30, 2.20)	0.68
>1000 (exceeds)	91	1.00	0.17 (0.01, 2.58)	0.20	15 (2.20, 98)	0.01
Interaction term p-value			0.86		0.22	

MIREC: Maternal-Infant Research on Environmental Chemicals Study, DMA: dimethylarsinic acid, BMI: Body Mass Index, OR: Odds Ratio

[a] Models adjusted for maternal age at delivery (continuous), maternal birth country (Canada v. foreign), prenatal multivitamin use (yes v. no), maternal depressive symptoms (continuous CES-D scores), maternal pre-pregnancy BMI (normal/underweight v. overweight v. obese), income (continuous), and parity (nulliparous v. 1 previous child v. 2+ previous children).

[b] Anthropometric Profile: Average Adiposity (n=218) are characterized by average adiposity, serves as the reference group

[c] Anthropometric Profile: Low Adiposity: (n=41) are characterize by less adiposity

[d] Anthropometric Profile: Excess Adiposity (n=105) are characterized by increased adiposity.

[e] Total folate: sum of the five folate vitamers, 5-methylTHF, folic acid, tetrahydrofolate (THF), 5-formylTHF, and 5,10-methenylTHF

[f] Daily folic acid intake from supplements based on maternal self-report on standardized questionnaires, note this information was collected at only one time point, after the 1st trimester baseline visit

[g] Note, we excluded participants who did not meet daily recommendations for folic acid intake from supplements due to small sample size, resulting in a subsample of n=345 participants. Within this subsample, n=203 participants were included in the Average Adiposity anthropometric profile, n=40 were included in the Low Adiposity profile, and n=102 were included in the Excess Adiposity profile.

Figure S1. Flow chart of participant selection to final sample size

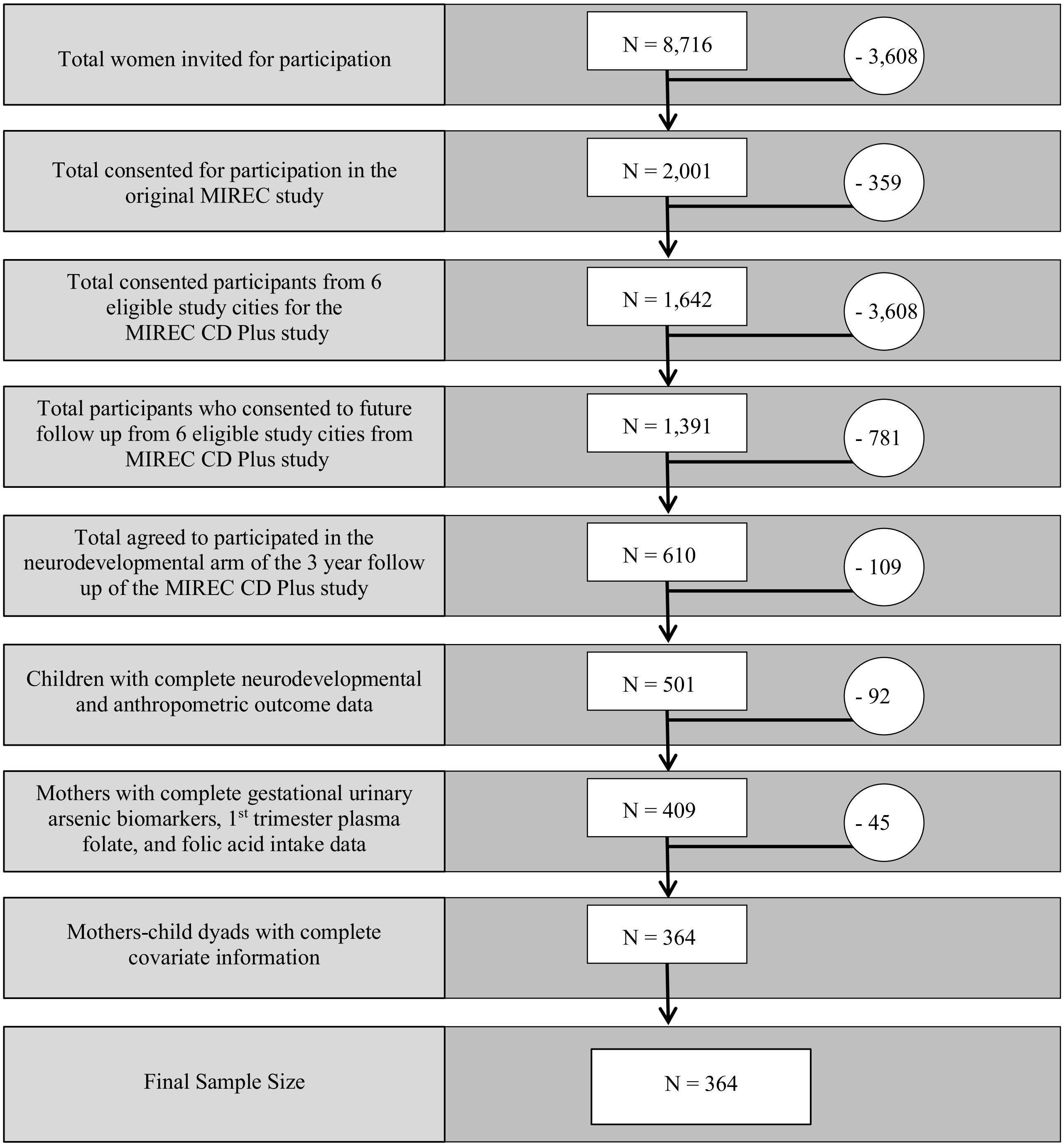

Figure S2. Directed Acyclic Graph used to select covariates in the association between gestational arsenic exposure and child health outcomes, adjusting for: Maternal Race (Canadian born v. foreign born), annual household income, prenatal vitamin use, pre-pregnancy BMI, maternal depressive symptoms (CES-D scores), maternal age, and parity

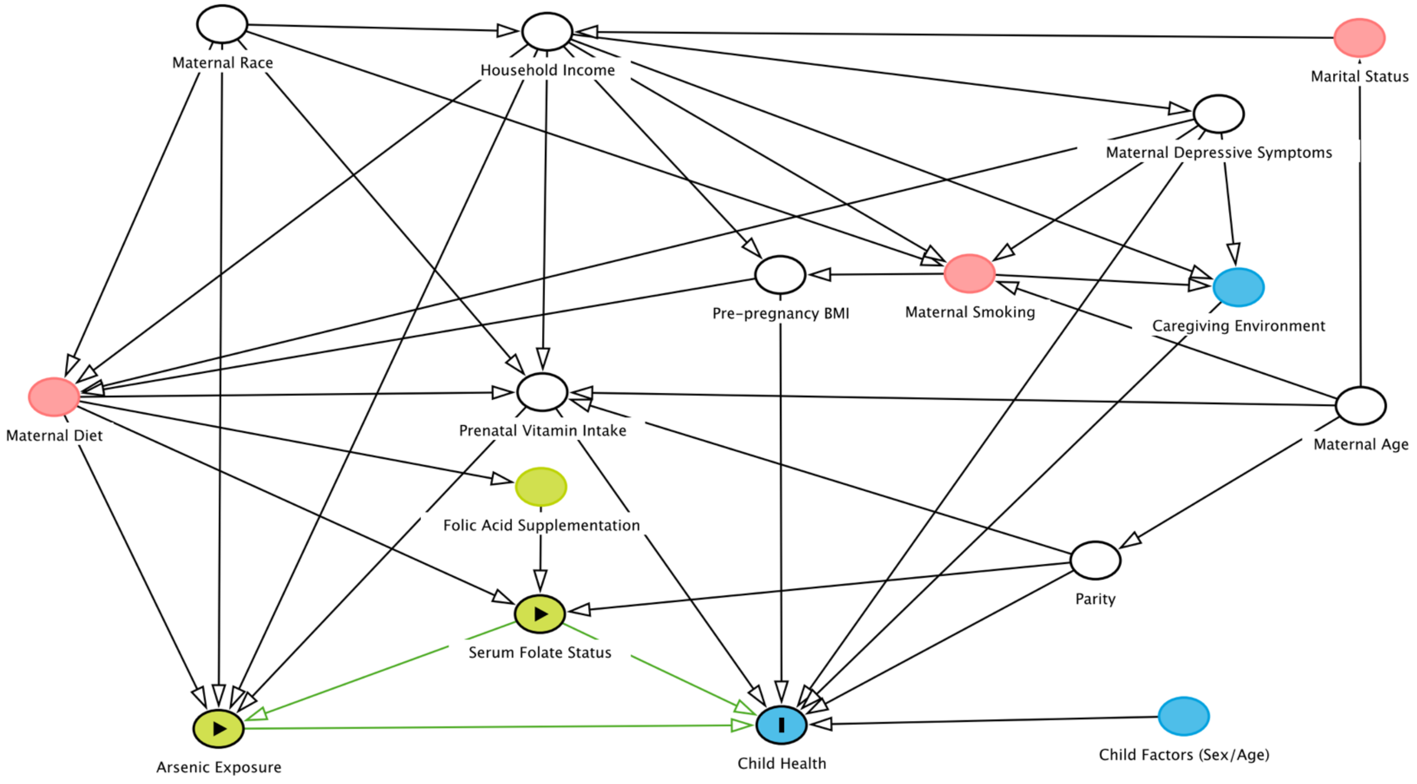

**Key:**	Exposure	Outcome	Ancestor of exposure	Ancestor of outcome	Ancestor of exposure and outcome	Adjusted variable	Causal path	Biased path	Unbiased path
	▶	▌							

Figure S3. Pearson correlation coefficients among plasma folate biomarkers (nmol/L) and maternal 1st trimester urinary arsenic concentrations (μg As/L) among MIREC Study participants

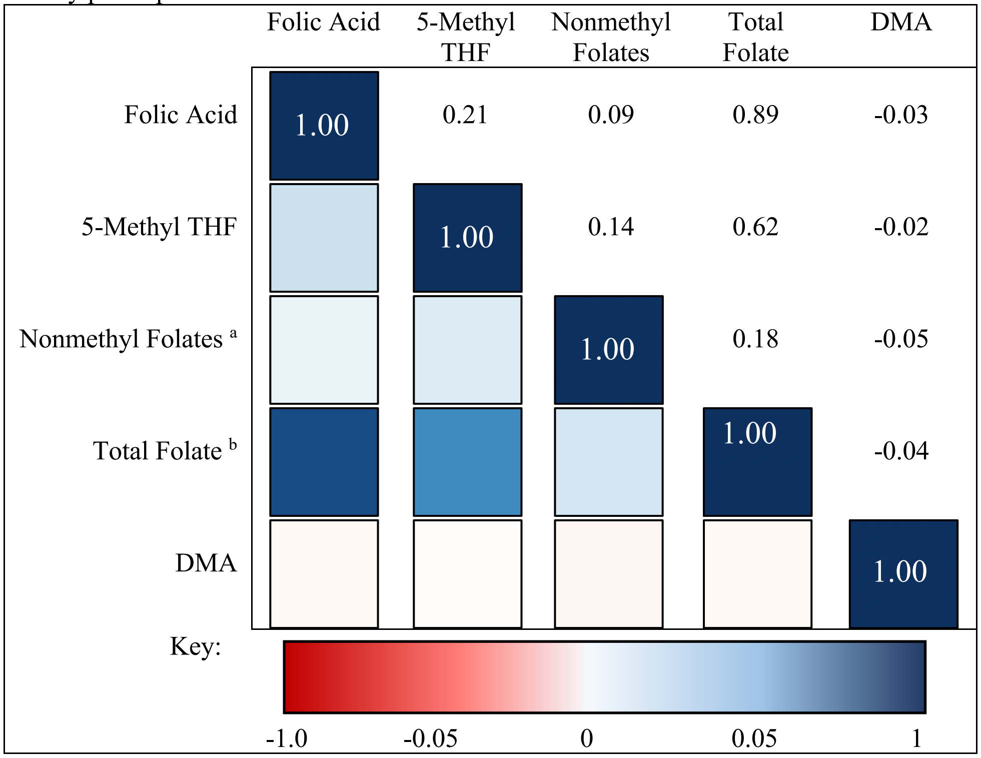

MIREC: Maternal-Infant Research on Environmental Chemicals Study, DMA: dimethylarsinic acid, Nonmethyl folates: sum of THF, 5-FormylTHF, and 5,10-MethenylTHF. Total folates: sum of five folate vitamers: 5-MethylTHF, folic acid, tetrahydrofolate [THF], 5-FormylTHF, 5,10-MethenylTHF).
